# Clinical Profile and Morbidity Patterns in Neonates Born Through Meconium-Stained Amniotic Fluid Based on a Prospective Observational Study From a Rural Tertiary Care Center in India

**DOI:** 10.7759/cureus.90332

**Published:** 2025-08-17

**Authors:** Gaurav Dutta, Suresh Kumar Yadav, Ekansh Rathoria, Rohitash Lahari, Akash Srivastava, Richa Rathoria

**Affiliations:** 1 Pediatrics, Hind Institute of Medical Sciences, Sitapur, IND; 2 Obstetrics and Gynecology, Hind Institute of Medical Sciences, Sitapur, IND

**Keywords:** acute kidney injury, apgar score, hypoxic-ischemic encephalopathy, meconium aspiration syndrome, meconium-stained amniotic fluid, neonatal morbidity, neonatal mortality, perinatal asphyxia, persistent pulmonary hypertension of the newborn, resuscitation

## Abstract

Background

Meconium observed in the amniotic fluid during delivery may warrant clinical monitoring due to potential neonatal complications. This study aimed to assess the incidence, risk factors, and outcomes associated with meconium aspiration syndrome (MAS) in neonates born through meconium-stained amniotic fluid (MSAF) at a rural tertiary care center in India.

Materials and methods

A prospective observational study was conducted over 18 months in the neonatal intensive care unit (NICU) of a rural tertiary hospital. A total of 126 inborn neonates with MSAF were enrolled. Maternal and neonatal demographic, clinical, and outcome data were collected. Statistical analyses included chi-square tests and Pearson’s correlation for univariate associations, and multivariable logistic regression was used to identify independent predictors of MAS and related outcomes. A p-value <0.05 was considered statistically significant.

Results

The incidence of MAS among neonates born through MSAF was 31.8% (40/126). The independent predictors of MAS included multigravida status (odds ratio [OR] = 3.633; p = 0.012), post-term gestation (OR = 4.126; p = 0.040), and maternal comorbidities (OR = 0.189; p = 0.003). MAS significantly predicted the need for resuscitation (OR = 0.038; p < 0.001), oxygen therapy (OR = 0.009; p < 0.001), shock (OR = 0.023; p < 0.001), acute kidney injury (AKI) (OR = 0.089; p < 0.001), persistent pulmonary hypertension of the newborn (PPHN) (OR = 0.019; p < 0.001), and hypoxic-ischemic encephalopathy (HIE) (OR = 0.028; p < 0.001). Abnormal creatinine-phosphokinase myocardial band (CPK-MB) levels (OR = 34.0; p < 0.001), abnormal chest X-ray findings (OR = 64.8; p < 0.001), and increased NICU stay (OR = 0.129; p < 0.001) were also strongly associated. Neonatal mortality was significantly higher among MAS cases (OR = 4.703; p = 0.009).

Conclusion

MAS remains a significant contributor to neonatal morbidity and mortality in low-resource settings. Early identification of at-risk neonates, improved targeted intrapartum monitoring, and timely resuscitative efforts are crucial. Integration of standardized MAS risk assessment protocols, strengthening emergency obstetric and newborn care (EmONC) infrastructure, and implementing regular staff training at all healthcare levels are urgently needed. Multicentric validation studies should also be prioritized to guide evidence-based interventions and reduce MAS-related complications in low-resource settings.

## Introduction

Meconium-stained amniotic fluid (MSAF) is a significant clinical concern in obstetrics and neonatology due to its association with adverse perinatal outcomes [1]. The presence of meconium in the amniotic fluid is observed in approximately 4-22% of all deliveries and is often considered an indicator of fetal maturity or intrauterine distress [2]. In utero meconium passage is uncommon before 34 weeks but becomes increasingly frequent in term and post-term fetuses, particularly in the presence of fetal distress such as hypoxia, infection, or cord compression, which may lead to its aspiration [3].

Meconium aspiration syndrome (MAS) is defined as respiratory distress in neonates born through MSAF, not attributable to other causes, and typically associated with characteristic radiological features [2]. MAS affects approximately 5-20% of neonates born through MSAF; however, certain studies in high-risk settings have reported incidences as high as 35% [4,5]. The mortality rate for MAS is estimated to range between 5% and 12% [3].

MAS is characterized by respiratory distress shortly after birth, often accompanied by tachypnea, grunting, retractions, hypoxemia, and chest radiograph findings such as patchy infiltrates, hyperinflation, atelectasis, and air leaks, features consistent with chemical pneumonitis and airway obstruction [2,5].

The pathophysiology of MAS involves meconium-induced airway obstruction, inflammation, and surfactant inactivation, often leading to complications such as pneumothorax, hypoxic-ischemic encephalopathy (HIE), and persistent pulmonary hypertension of the newborn (PPHN), which is associated with high mortality and confirmed by echocardiography showing shunting and elevated pulmonary pressures [6,7].

In severe cases, MAS may lead to systemic complications such as HIE [characterized by altered consciousness, seizures, and abnormal electroencephalogram (EEG), and classified by Sarnat staging], myocardial dysfunction [indicated by edema, third space fluid accumulation, hepatomegaly, S3 gallop, cardiogenic shock, poor perfusion (capillary refill time >3 seconds), hypotension, and elevated creatinine-phosphokinase myocardial band (CPK-MB) >75 IU/L], acute kidney injury (AKI) [classified by the modified kidney disease improving global outcomes (KDIGO) criteria based on serum creatinine rise and reduced urine output], as well as sepsis, shock, and polycythemia (hematocrit >65% or hemoglobin >22 g/dL), all contributing to increased neonatal morbidity and mortality [8-17].

In recent years, changes in neonatal resuscitation guidelines have shifted clinical practice away from routine endotracheal suctioning for non-vigorous neonates with MSAF [18]. Instead, individualized supportive management, including supplemental oxygen, continuous positive airway pressure (CPAP), invasive mechanical ventilation (IMV), surfactant therapy, and inhaled nitric oxide, is now recommended based on the infant’s respiratory status [19].

Despite advancements in neonatal care, MAS continues to be a major contributor to neonatal mortality in low- and middle-income countries (LMICs) like India, especially in rural settings, due to delayed diagnosis and referrals, limited resuscitation skills, inadequate intrapartum care, and poor access to intensive care [20]. Region-specific data from tertiary centers are crucial to understanding the epidemiology, risk factors, and outcomes of MSAF and MAS in these settings [9].

Sustainable Development Goal (SDG) 3.2 targets a reduction in neonatal mortality to 12 or fewer deaths per 1,000 live births in all countries by the year 2030 [21]. Under the India Newborn Action Plan (INAP) 2014, India has pledged to achieve a single-digit stillbirth rate (SBR) and newborn mortality rate (NMR) by 2030 [22]. In alignment with these national and global health goals, this study addresses the burden and predictors of MAS, thereby contributing evidence to inform targeted perinatal interventions in resource-constrained settings.

While several global studies have addressed the incidence and risk factors of MAS, recent data from rural Indian neonatal intensive care units (NICU) remain limited [1,4,8,9,11-13]. Moreover, little has been published on the integrated analysis of maternal and neonatal predictors of MAS and associated outcomes using multivariable models in such settings. This study aimed to evaluate the morbidity and mortality patterns among neonates born with MSAF and to identify maternal and neonatal factors associated with MAS and adverse neonatal outcomes.

## Materials and methods

This hospital-based, prospective observational study was conducted over 18 months from August 2023 to January 2025 in the NICU of the Department of Pediatrics, Hind Institute of Medical Sciences, Ataria, Sitapur, Uttar Pradesh, India, which serves as a referral tertiary care hospital catering to a semi-urban and rural population. Ethical approval was taken from the Institutional Ethics Committee (IEC) of Hind Institute of Medical Sciences, and informed consent was obtained from parents or legal guardians before the enrolment of neonates.

The study included all neonates with a documented history of MSAF at birth, admitted to the NICU within the first 24 hours of life. The consistency of MSAF was classified by the attending obstetricians at the time of delivery as either thin (lightly stained, watery appearance) or thick (viscous, pea-soup-like) based on visual inspection, per standard clinical protocols [20]. Neonates born between 34 and 37 completed weeks were included as late preterm infants, as this group is developmentally mature enough to undergo standardized respiratory evaluation and management.

We excluded neonates <34 weeks of gestation to minimize confounding from prematurity-related complications, such as surfactant deficiency and intraventricular hemorrhage, which may independently influence respiratory outcomes and Apgar scores. Additional exclusion criteria included neonates with major congenital anomalies or chromosomal abnormalities, outborn deliveries, stillbirths, incomplete or missing medical records, intrauterine infections (e.g., TORCH infections), and cases in which the diagnosis of MAS was not confirmed clinically and radiologically.

A consecutive sampling method was employed, wherein all inborn neonates with MSAF who met the inclusion criteria during the study period were enrolled. The sample size (n0 = 113) was calculated using the Cochran formula for infinite population (n0 = z2.p.q/e2), which was then adjusted using modified Cochran formula for small populations [n1 = n0/[1+{(n0−1)/N}]] to get a adjusted sample size (n1) of 105 neonates, taking population size (N) as 1500, z as 1.96 (for 95% confidence interval), p as 0.0796 based on a reported 7.96% prevalence of MAS as per previous study, q (q=1-p) as 0.9204, with a margin of error (e) of 0.05 [23,24]. Accounting for a 20% non-response or dropout rate, the final sample size (n) was adjusted to 126 neonates.

Data were recorded using a structured and pre-tested proforma, which included systematically classified maternal and neonatal information, clinical findings, and outcome parameters. Maternal variables included age (categorized as ≤25 years and >25 years), gravida (primigravida or multigravida), gestational age determined by last menstrual period (LMP) and early ultrasound (classified as preterm <37 weeks, term 37-<42 weeks, and post-term ≥42 weeks), mode of delivery [normal vaginal delivery (NVD) or lower-segment cesarean section (LSCS)], and the presence or absence of maternal comorbidities such as anemia, antepartum hemorrhage (APH), gestational diabetes mellitus (GDM), hypertensive disorders of pregnancy (HDP), intrahepatic cholestasis of pregnancy (ICP), oligohydramnios, polyhydramnios, and premature rupture of membranes (PROM). The maternal age categories were selected post hoc based on the age range observed in our study population (18-34 years), as no mothers were aged ≥35 years; hence, the conventional cutoff for advanced maternal age could not be applied.

Neonatal variables included gender, birth weight (categorized as <1500 g, 1500-<2500 g, and ≥2500 g), APGAR scores at 1 and 5 minutes (classified as <7 or ≥7), need for resuscitation (yes/no), and type of oxygen therapy administered (none, nasal prongs, CPAP, or IMV). Neonatal investigations included complete blood count (CBC), C-reactive protein (CRP), blood culture, sepsis screen, CPK-MB, serum electrolytes, and renal function tests. A chest radiograph was done for respiratory evaluation, while 2D-echocardiography was done when PPHN was suspected. Cranial ultrasound and EEG were done in cases with neurological involvement.

Outcome variables (dependent variables) included the occurrence of MAS, presence of specific neonatal morbidities such as shock, PPHN, AKI, seizures, polycythemia, and HIE (which was staged using Sarnat staging), all recorded as present or absent [15]. Perinatal asphyxia was defined by APGAR scores <7 at 1 and 5 minutes [25]. Additionally, investigation parameters included results of the sepsis screen (positive/negative), levels of CPK-MB (normal/abnormal), and chest radiograph findings (normal/abnormal). The duration of NICU stay was categorized as <7 days or ≥7 days, and the final clinical outcome was recorded as either discharge or death. These classifications were intended to enhance the clarity and consistency of data analysis and interpretation. To minimize inter-observer variability, all neonatal diagnoses, including MAS, PPHN, AKI, and HIE, were made using standardized clinical and radiological criteria, and assessments were performed by trained neonatologists following departmental protocols.

Statistical analysis

Data were compiled using Microsoft Excel (Microsoft, Redmond, WA) and subsequently analyzed with IBM SPSS Statistics for Windows, Version 26.0 (IBM Corp., Armonk, NY). Categorical variables were summarized as frequencies and percentages, while continuous variables were presented as means with standard deviations (mean ± SD). The chi-square test was employed to assess associations between categorical variables, and Pearson’s correlation coefficient (r) was used to evaluate linear relationships between continuous variables. Binary logistic regression was utilized to determine the predictors of MAS and to assess its association with various clinical morbidities. A p-value of less than 0.05 was considered statistically significant.

## Results

Maternal age ranged from 18 to 34 years (mean 24.03 ± 3.26), with 65.9% (83/126) aged ≤25 years and 55.6% (70/126) being primigravida. Most deliveries occurred at term (60.3%, 76/126), with 27% (34/126) preterm and 12.7% (16/126) post-term. LSCS was performed in 56.3% (71/126), and 58.7% (74/126) of mothers had comorbidities, mainly anemia (18.3%, 23/126), HDP (15.1%, 19/126), and PROM (6.3%, 8/126). The mean neonatal birth weight was 2627.8 ± 617.1 g; 69% (87/126) were male, and 55.5% (70/126) weighed >2500 g.

MAS was significantly associated with multigravidity (χ² = 12.616, p < 0.0001; r = 0.316), post-term pregnancies (χ² = 9.461, p = 0.009), vaginal delivery (χ² = 4.570, p = 0.033; r = -0.190), and maternal comorbidities (χ² = 20.013, p < 0.0001; r = 0.399), indicating moderate positive correlations with multigravidity and comorbidities (Table 1). No significant association was found between MAS and maternal age (χ² = 0.899, p = 0.343; r = 0.084), neonatal gender (χ² = 0.025, p = 0.875; r = 0.014), or birth weight (χ² = 2.785, p = 0.248; r = -0.134). Although gestational age showed a significant chi-square result, the correlation was not statistically significant (r = 0.103, p = 0.250).

**Table 1:** Association of MAS With Maternal and Neonatal Characteristics in MSAF Cases (n = 126) Data presented as n (%), significant p-value <0.05. MAS: meconium aspiration syndrome; NVD: normal vaginal delivery; LSCS: lower-segment caesarian section; MSAF: meconium-stained amniotic fluid.

Characteristics	Category	MAS		Chi-square test (χ²)	p-Value (χ²)	Pearson’s r	p-Value (r)
		No (n = 86)	Yes (n = 40)				
Maternal age (years)	≤25	59 (68.6)	24 (60)	0.899	0.343	0.084	0.347
	>25	27 (31.4)	16 (40)				
Gravida	Primigravida	57 (66.3)	13 (32.5)	12.616	<0.0001	0.316	<0.0001
	Multigravida	29 (33.7)	27 (67.5)				
Gestational age (weeks)	<37	22 (25.6)	12 (30)	9.461	0.009	0.103	0.250
	37-<42	58 (67.4)	18 (45)				
	>42	6 (7)	10 (25)				
Mode of delivery	NVD	32 (37.2)	23 (57.5)	4.570	0.033	-0.19	0.033
	LSCS	54 (62.8)	17 (42.5)				
Maternal comorbidities	Present	39 (45.3)	35 (87.5)	20.013	<0.0001	0.399	<0.0001
	Absent	47 (54.7)	5 (12.5)				
Gender of neonate	Male	59 (68.6)	28 (70)	0.025	0.875	0.014	0.876
	Female	27 (31.4)	12 (30)				
Birth weight of neonate (grams)	<1500	1 (1.2)	2 (5)	2.785	0.248	-0.134	0.136
	1500-<2500	34 (39.5)	19 (47.5)				
	>2500	51 (59.3)	19 (47.5)				

The mean APGAR scores at 1 and 5 minutes were 7.29 ± 2.14 and 8.37 ± 1.51, respectively, with ≥7 scores observed in 65.9% (83/126) and 88.1% (111/126) of neonates. Resuscitation and oxygen therapy were required in 32.5% (41/126) and 31.8% (40/126), respectively; among those needing oxygen, 15% (6/40) received nasal prongs/hood, 50% (20/40) CPAP, and 35% (14/40) IMV.

MAS developed in 31.8% (40/126) of neonates and was significantly associated with 1-minute APGAR <7 (72.5% vs. 16.3%, χ² = 38.386, p < 0.0001; r = 0.347), but not with 5-minute scores (p = 0.888) (Table 2). MAS cases had significantly higher rates of resuscitation (77.5% vs. 11.6%, χ² = 53.967, p < 0.0001; r = -0.219) and oxygen support (87.5% vs. 5.8%, χ² = 84.075, p < 0.0001; r = -0.319), indicating early postnatal compromise. MAS was also strongly associated with shock (40.5%, χ² = 59.658, p < 0.0001; r = 0.688), AKI (21.4%, χ² = 28.414, p < 0.0001; r = 0.475), and PPHN (19%, χ² = 49.127, p < 0.0001; r = 0.624). Significant associations were noted for seizures (χ² = 9.963, p = 0.002), positive sepsis screen (46%, χ² = 31.374, p < 0.0001; r = 0.499), abnormal CPK-MB levels (42.9%, χ² = 53.184, p < 0.0001; r = -0.650), and abnormal chest radiographs (χ² = 72.441, p < 0.0001; r = -0.758). Polycythemia showed no significant association (p = 0.438). HIE occurred in 27.8% (35/126), with MAS significantly linked to moderate and severe stages (χ² = 58.427, p < 0.0001; r = 0.681). Longer NICU stays (≥7 days) were more frequent in MAS cases (χ² = 24.724, p < 0.0001; r = 0.443), and mortality was significantly higher among MAS neonates (22.5% vs. 5.8%; χ² = 7.696, p = 0.006; r = -0.247), underscoring the adverse outcomes associated with MAS.

**Table 2:** Association of MAS With Morbidities, Investigations, and Outcomes in Neonates With MSAF (n = 126) Data presented as n (%), significant p-value <0.05. MSAF: meconium-stained amniotic fluid; MAS: meconium aspiration syndrome; AKI: acute kidney injury; PPHN: persistent pulmonary hypertension; CPK-MB: creatine phosphokinase myocardial band; HIE: hypoxic ischemic encephalopathy.

Neonatal parameters	Category	MAS		Chi-square test (χ²)	p-Value (χ²)	Pearson’s r	p-Value (r)
		No (n = 86)	Yes (n = 40)				
APGAR at 1 minute	<7	14 (16.3)	29 (72.5)	38.386	<0.0001	0.347	<0.0001
	≥7	72 (83.7)	11 (27.5)				
APGAR at 5 minutes	<7	10 (11.6)	5 (12.5)	0.020	0.888	0.145	0.104
	≥7	76 (88.4)	35 (87.5)				
Resuscitation	Not required	76 (88.4)	9 (22.5)	53.967	<0.0001	-0.219	0.014
	Required	10 (11.6)	31 (77.5)				
Oxygen support	Not required	81 (94.2)	5 (12.5)	84.075	<0.0001	-0.319	<0.0001
	Required	5 (5.8)	35 (87.5)				
Shock	No	71 (82.6)	4 (10)	59.658	<0.0001	0.688	<0.0001
	Yes	15 (17.4)	36 (90)				
AKI	No	79 (91.9)	20 (50)	28.414	<0.0001	0.475	<0.0001
	Yes	7 (8.1)	20 (50)				
PPHN	No	84 (97.7)	18 (45)	49.12	<0.0001	0.624	<0.0001
	Yes	2 (2.3)	22 (55)				
Seizures	No	85 (98.8)	34 (85)	9.963	0.002	0.281	0.001
	Yes	1 (1.2)	6 (15)				
Polycythemia	No	79 (91.9)	35 (87.5)	0.602	0.438	0.069	0.442
	Yes	7 (8.1)	5 (12.5)				
Sepsis screen	Negative	61 (71)	7 (17.5)	31.374	<0.0001	0.499	<0.0001
	Positive	25 (29)	33 (82.5)				
CPK-MB	Abnormal	18 (20.9)	36 (90)	53.184	<0.0001	-0.650	<0.0001
	Normal	68 (79.1)	4 (10)				
Chest radiograph findings	Abnormal	5 (5.8)	32 (80)	72.441	<0.0001	-0.758	<0.0001
	Normal	81 (94.2)	8 (20)				
HIE	No	80 (93)	11 (27.5)	58.427	<0.0001	0.681	<0.0001
	Yes	6 (7)	29 (72.5)				
Duration of hospital stay	<7 days	62 (72.1)	10 (25)	24.724	<0.0001	0.443	<0.0001
	≥7 days	24 (27.9)	30 (75)				
Outcome	Discharge	81 (94.2)	31 (77.5)	7.696	0.006	-0.247	0.005
	Death	5 (5.8)	9 (22.5)				

In multivariable logistic regression analysis, multigravida status (odds ratio [OR] = 3.633, 95% CI: 1.33-9.924, p = 0.012) and post-term gestation (OR = 4.126, 95% CI: 1.065-15.983, p = 0.040) were found to be significant independent predictors of MAS (Table 3). Additionally, maternal comorbidities were significantly associated with increased MAS risk (OR = 0.189, 95% CI: 0.062-0.571, p = 0.003). The mode of delivery showed borderline statistical significance (p = 0.064), suggesting LSCS may reduce MAS risk. Other variables, including maternal age, newborn gender, and birth weight, were not significantly associated with MAS.

**Table 3:** Multivariable Logistic Regression Analysis for Predictors of MAS (n = 126) Statistically significant predictors (p < 0.05). B: regression coefficient; SE: standard error; Wald: Wald chi-square test statistic; OR: odds ratio; CI: confidence interval; GA: gestational age; LSCS: lower-segment cesarean section; NVD: normal vaginal delivery.

Predictor variable	B	SE	Wald	p-Value	OR (Exp(B))	95% CI for OR
Maternal age >25 years	–0.207	0.508	0.166	0.683	0.813	0.301-2.198
Multigravida (vs. Primigravida)	1.290	0.513	6.331	0.012	3.633	1.330-9.924
GA: Post-term (vs. Term)	1.417	0.691	4.207	0.040	4.126	1.065-15.983
GA: Preterm (vs. Term)	0.362	0.585	0.384	0.536	1.437	0.457-4.522
Mode of delivery (LSCS vs. NVD)	–0.944	0.509	3.437	0.064	0.389	0.143-1.055
Maternal comorbidities (Yes vs. No)	–1.667	0.565	8.711	0.003	0.189	0.062-0.571
Gender (Female vs. Male)	–0.114	0.509	0.051	0.822	0.892	0.329-2.419
Birth weight: ≥2500 g (vs. 1500-2500 g)	–0.755	0.557	1.840	0.175	0.470	0.158-1.399
Birth weight: <1500 g (vs. 1500-2500 g)	0.570	1.356	0.176	0.674	1.768	0.124-25.236
Constant	–0.275	0.531	0.269	0.604	0.759	—

MAS was found to be a statistically significant predictor for several morbidities, including the need for resuscitation (OR = 0.038, p < 0.001), oxygen therapy (OR = 0.009, p < 0.001), shock (OR = 0.023, p < 0.001), AKI (OR = 0.089, p < 0.001), PPHN (OR = 0.019, p < 0.001), seizures (OR = 0.067, p = 0.014), and HIE (OR = 0.028, p < 0.001) (Table 4). MAS was also associated with abnormal CPK-MB levels (OR = 34.0, p < 0.001), chest radiograph abnormalities (OR = 64.8, p < 0.001), and longer NICU stay (OR = 0.129, p < 0.001). Additionally, MAS significantly increased the odds of neonatal mortality (OR = 4.703, p = 0.009). These findings reinforce MAS as a strong predictor of both respiratory and systemic complications in affected neonates.

**Table 4:** Binary Logistic Regression Analysis Showing MAS as a Predictor of Neonatal Morbidity and Mortality (n = 126) Reference category for all binary comparisons is "No MAS". Significant p-value <0.05. B: unstandardized regression coefficient; SE: standard error; Wald: Wald chi-square test statistic; Exp(B): odds ratio; CI: confidence interval; AKI: acute kidney injury; PPHN: persistent pulmonary hypertension; CPK-MB: creatine phosphokinase-MB; HIE: hypoxic ischemic encephalopathy.

Dependent variable	B	SE	Wald	p-Value	Exp(B)	95% CI for Exp(B)
Resuscitation required	–3.265	0.506	41.554	<0.001	0.038	0.014–0.103
	1.237	0.379	10.669	0.001	3.444	—
Oxygen therapy type	-4.731	0.664	50.762	<0.001	0.009	0.002-0.032
	1.946	0.478	16.566	<0.001	7.000	—
1-Minute APGAR score	2.607	0.459	32.255	<0.001	13.558	5.514-33.338
	–0.969	0.354	7.494	0.006	0.379	—
5-Minute APGAR score	0.082	0.585	0.020	0.888	1.086	0.345-3.414
	1.946	0.478	16.566	<0.001	7.000	—
Shock	-3.752	0.599	39.262	<0.001	0.023	0.007-0.076
	2.197	0.527	17.380	<0.001	9.000	–
AKI	-2.424	0.505	22.987	<0.001	0.089	0.033-0.239
	0.000	0.316	0.000	1.000	1.000	–
PPHN	-3.938	0.783	25.306	<0.001	0.019	0.004-0.090
	0.201	0.318	0.399	0.528	1.222	–
Seizures	-2.708	1.099	6.072	0.014	0.067	0.008-0.575
	-1.735	0.443	15.345	0.000	0.176	–
Polycythemia	-0.478	0.620	0.594	0.441	0.620	0.184-2.090
	-1.946	0.478	16.566	0.000	0.143	–
Sepsis screen positive	-2.443	0.479	25.991	<0.001	0.087	0.034-0.222
	1.551	0.416	13.885	<0.001	4.714	–
Abnormal CPK-MB levels	3.526	0.590	35.729	<0.001	34.000	10.698-108.055
	-2.197	0.527	17.380	<0.001	0.111	–
Abnormal chest radiograph	4.171	0.607	47.206	<0.001	64.800	19.715-212.989
	-1.386	0.395	12.300	<0.001	0.250	–
HIE	-3.560	0.552	41.605	<0.001	0.028	0.010-0.084
	0.969	0.354	7.494	0.006	2.636	–
Duration of stay	-2.048	0.437	21.938	<0.001	0.129	0.055-0.304
	1.099	0.365	9.052	0.003	3.000	–
Outcome	1.548	0.596	6.739	0.009	4.703	1.461-15.138
	1.237	0.379	10.669	0.001	3.444	—

## Discussion

MSAF continues to be a significant clinical concern due to its association with neonatal morbidity and mortality, particularly in LMICs. In the present prospective observational study conducted at a rural tertiary care center in Northern India, the incidence of MAS among neonates born through MSAF was found to be 31.8%, which is substantially higher than the 5-20% reported in global literature [1,2]. Some regional studies have reported similar or even higher rates, particularly in resource-limited settings, likely due to delayed access to skilled obstetric care and neonatal resuscitation [4,5].

Multigravida status emerged as a strong maternal predictor of MAS (p < 0.0001), consistent with the findings by Patel et al. [26]. Multivariable logistic regression revealed that multigravida status was a significant independent predictor of MAS (OR = 3.633; p = 0.012), aligning with prior research linking higher parity to increased fetal stress and meconium passage [26].

Post-term gestation (>42 weeks) was also significantly associated with MAS (p = 0.009), aligning with the evidence that delayed deliveries increase the risk of fetal hypoxia and meconium passage [3,18]. Post-term gestation was also significantly associated with MAS (OR = 4.126; p = 0.040), consistent with literature indicating prolonged pregnancies contribute to placental insufficiency and hypoxia [3,5,18].

A higher incidence of MAS was observed in neonates delivered vaginally (23/40, 57.5%) compared to LSCS (17/40, 42.5%) (p = 0.033), consistent with the findings by Tanveer et al., which support earlier studies suggesting that emergency cesarean deliveries may reduce aspiration risk when performed promptly in MSAF cases [27]. Although vaginal delivery showed a borderline association with higher MAS risk (p = 0.064), cesarean section appeared protective, likely due to earlier intervention in high-risk labors [27].

Maternal comorbidities such as anemia, hypertensive disorders, and PROM were present in 87.5% (35/40) of MAS cases versus 45.3% (39/86) in the non-MAS group (p < 0.0001), indicating that maternal health plays a pivotal role in the pathogenesis of MAS, as also reported by Moolchandani et al. [24]. Additionally, the presence of maternal comorbidities such as hypertensive disorders and anemia significantly increased MAS risk (OR = 0.189; p = 0.003), corroborating earlier findings that compromised maternal health can predispose to adverse neonatal outcomes [5,24].

Neonatal factors such as birth weight and gender were not significantly associated with MAS in our study (p > 0.05). However, Tanveer et al. reported a higher likelihood of MAS in neonates weighing ≤3 kg (AOR: 0.39; 95% CI: 0.19-0.85) and in females (AOR: 1.89; 95% CI: 0.91-3.95), indicating potential demographic variability in risk [27]. No significant associations were found with maternal age, birth weight, or neonatal gender, diverging from certain regional studies that reported conflicting results [24].

A significantly higher proportion of MAS cases had APGAR scores <7 at 1 minute (29/40, 72.5%), consistent with the findings by Monfredini et al. and Moin et al., who emphasized the predictive value of early neonatal depression for MAS development [2,10]. A systematic review and meta-analysis conducted by Luo et al. identified a 5-minute Apgar score of less than 7 as the most significant risk factor for MAS, with a pooled OR of 14.89 (95% CI: 9.52-23.28; p < 0.001) across eight studies [28].

The requirement for resuscitation and oxygen therapy was markedly higher in MAS neonates [77.5% (31/40) and 87.5% (35/40), respectively], reflecting greater respiratory compromise, which aligns with earlier studies indicating that most MAS cases necessitate respiratory support, including CPAP or mechanical ventilation [3,10,18,19]. MAS was independently associated with the need for resuscitation (OR = 0.038), oxygen therapy (OR = 0.009), and shock (OR = 0.023), all with highly significant p-values (p < 0.001), indicating the severe initial compromise these neonates face.

Severe complications such as shock, PPHN, and AKI were also significantly more common in the MAS group (p < 0.0001), corroborating prior research that identified MAS as a driver of systemic inflammation and hypoxic injury [7,9,10]. HIE was present in 72.5% (29/40) of MAS neonates, in agreement with studies highlighting the association between MAS, perinatal asphyxia, and subsequent neurological injury [14]. Furthermore, MAS significantly predicted the occurrence of AKI (OR = 0.089), PPHN (OR = 0.019), and HIE (OR = 0.028), reflecting its multisystemic impact. These findings are in concordance with previous studies, which highlight the cascade of organ dysfunction resulting from perinatal hypoxia and aspiration-related inflammation [5,6,8,12,15].

Elevated CPK-MB levels and abnormal chest radiograph findings [90% (36/40) and 80% (32/40), respectively] were strongly linked with MAS, consistent with prior evidence of myocardial strain and radiological patterns of chemical pneumonitis in these neonates [10,11]. The elevated odds of abnormal CPK-MB levels (OR = 34.0) and chest radiograph changes (OR = 64.8) suggest significant myocardial and pulmonary involvement in MAS, also emphasized by earlier reports [10,11]. MAS was significantly associated with positive sepsis screens (OR = 4.714), possibly due to impaired pulmonary clearance and compromised immunity in these neonates [9].

Furthermore, MAS cases had significantly longer NICU stays [≥7 days in 75% (30/40)] and higher mortality [22.5% (9/40)] compared to non-MAS neonates, which was consistent with the Moin et al. study, underscoring the severity and clinical burden of the condition [10]. This pattern reflects findings in LMICs, where limited access to neonatal intensive care and delayed interventions contribute to elevated MAS-related morbidity and mortality [20]. A prolonged NICU stay ≥7 days (OR = 0.129) and higher mortality (OR = 4.703) among MAS cases further reaffirm its grave clinical trajectory, consistent with regional and global data from similar resource-limited settings [2,10,13]. These findings reinforce the need for prompt identification and aggressive management of MAS to reduce systemic complications and fatal outcomes.

Strengths

It was a prospective observational study, which minimized recall bias and allowed for real-time, systematic data collection. The comprehensive analysis of both maternal and neonatal variables provided a holistic understanding of the multifactorial nature of MAS. Standardized diagnostic criteria were used for neonatal complications such as PPHN, HIE, and AKI, thereby enhancing diagnostic reliability. Furthermore, the application of multivariable logistic regression enabled the identification of independent predictors of MAS, adjusting for potential confounders. Importantly, the study was conducted in a rural tertiary care center in Northern India, making the findings especially relevant for low-resource healthcare settings that often encounter delays in obstetric and neonatal interventions.

Limitations

Despite its strengths, this study has some limitations. Being a single-center study conducted in a rural setup, the findings may not be generalizable to urban or high-resource healthcare settings. The exclusion of preterm neonates below 34 weeks and outborn infants may have resulted in underestimation of the full spectrum of MAS-related complications. In addition, long-term neurodevelopmental and respiratory outcomes were not assessed due to the lack of follow-up data. The relatively small sample size in certain subgroups may have limited the statistical power to detect associations, especially for rare complications. Finally, while logistic regression was performed, residual confounding from unmeasured variables cannot be entirely excluded.

## Conclusions

This study highlights the high incidence and clinical burden of MAS among neonates born through MSAF in a rural Indian setting. Multigravida status, post-term gestation, maternal comorbidities, and vaginal delivery emerged as significant independent predictors of MAS. Additionally, MAS was associated with serious neonatal complications, including PPHN, AKI, HIE, and increased mortality, along with prolonged NICU stays and greater need for resuscitation and respiratory support. These findings emphasize the need for improved intrapartum monitoring, early identification of high-risk pregnancies, and prompt neonatal resuscitation, particularly in resource-constrained environments. Strengthening neonatal intensive care capabilities and implementing standardized protocols for MAS management should be prioritized under national programs like the India Newborn Action Plan (INAP). Future research should include multicentric and longitudinal studies to validate findings and assess long-term outcomes in MAS survivors.
